# 2292 Evaluating the impact of a K-award on clinical and translational research

**DOI:** 10.1017/cts.2018.207

**Published:** 2018-11-21

**Authors:** Elias M. Samuels, Thomas E. Perorazio, Ellen Champagne, Brenda Eakin

**Affiliations:** University of Michigan School of Medicine

## Abstract

OBJECTIVES/SPECIFIC AIMS: Identify the impact of the provision of clinical and translational research training awards on investigators’ pursuit of clinical and translational research careers. METHODS/STUDY POPULATION: Propensity score matching and qualitative analysis/investigators receiving MICHR’s KL2 research training awards. RESULTS/ANTICIPATED RESULTS: While the evaluations of the impact of this service have shown participants find them to be valuable it is expected that participation in the workshop may be more beneficial to investigators with certain types of prior research experiences and who utilize more CTSA research support. DISCUSSION/SIGNIFICANCE OF IMPACT: Because this evaluation of a research service incorporate data representing investigator’s receipt of different CTSA resources, the findings can be used to inform the ongoing coordination of these services in ways that optimize their impact on the production of clinical and translational research. There is an enduring need for evaluations of CTSA programs to account for investigators’ use of different constellations of research services in order to identify what combinations of services over time are most effective at fostering successful clinical and translational research careers.

